# Comparative clinical utility of preoperative psoas muscle and skeletal muscle index cutoffs for risk stratification after esophagectomy

**DOI:** 10.1007/s00595-026-03433-7

**Published:** 2026-07-23

**Authors:** Hiroki Mizusawa, Osamu Shiraishi, Yuji Higashimoto, Masashi Shiraishi, Masaya Noguchi, Kengo Kanki, Honoka Natsume, Tomomi Tamura, Takushi Yasuda

**Affiliations:** 1 Department of Rehabilitation, Kindai University Hospital, 1-14-1 Mihara-dai, Minami-ku, Sakai, Osaka Japan; 2 Faculty of Medicine, Department of Rehabilitation Medicine, Kindai University, Osaka, Japan; 3 Faculty of Medicine, Department of Surgery, Kindai University, Osaka, Japan

**Keywords:** Esophagectomy, Psoas muscle index, Sarcopenia, Postoperative pulmonary complications, Survival

## Abstract

**Purpose:**

To compare the clinical utility of preoperative psoas muscle index (PMI) and skeletal muscle index (SMI) cutoff stratification for predicting postoperative pulmonary complications (PPCs) and survival after esophagectomy.

**Methods:**

This single-center retrospective study included 334 patients who underwent esophagectomy for thoracic esophageal cancer. Preoperative SMI and PMI were measured on CT images at the L3 level and classified using established cutoff values. Associations with PPCs were examined using multivariable logistic regression. The 5-year overall survival (OS) and recurrence-free survival (RFS) rates were evaluated.

**Results:**

PPCs occurred in 110 patients in the study. The low-PMI group had significantly higher rates of PPCs and pneumonia than the no low-PMI group, whereas no significant differences were observed between the SMI-based groups. In a multivariable analysis, a low PMI was independently associated with PPCs (OR 2.17, 95% CI 1.16–4.07; *p* = 0.02), but a low SMI was not. The low-PMI group showed worse 5-year OS and RFS in unadjusted analyses, whereas SMI was not associated with these outcomes. In Cox regression analyses adjusted for other prognostic factors, the association between a low PMI and long-term outcomes was attenuated.

**Conclusions:**

The preoperative PMI was more strongly associated with PPCs after esophagectomy than SMI. Although unadjusted analyses indicated an association, a low-PMI did not independently predict worse long-term outcomes in the adjusted analyses.

**Supplementary Information:**

The online version contains supplementary material available at https://doi.org/10.1007/s00595-026-03433-7.

## Introduction

Esophageal cancer is a leading cause of cancer-related death worldwide [1, 2]. Esophagectomy, the most invasive thoracic surgery, plays a substantial role in achieving locoregional control and offers the best chance of treating both localized and advanced esophageal cancer [3, 4]. The development of postoperative pulmonary complications (PPCs) after esophagectomy affects health-related quality of life, length of hospital stay, medical costs, and overall survival (OS) [5–8]. Preoperative risk factors for PPC development after esophagectomy include age, body mass index, smoking history, pulmonary and respiratory muscle function, and exercise tolerance [9]. Among the CT-based markers of sarcopenia, the skeletal muscle index (SMI) has been widely used for perioperative risk assessment. SMI, defined using the cutoff values proposed by Prado et al., has been reported to be associated with PPCs after esophagectomy [10, 11]. However, these thresholds have been proposed in studies involving European populations, and many Asian patients with esophageal cancer have scores below the cutoff values [12, 13]. Subsequently, Asian-specific cutoff values for low skeletal muscle mass have been proposed, reflecting the need for population-appropriate CT-based assessment of sarcopenia [14].

In parallel, the psoas muscle index (PMI) has attracted increasing attention as a simple CT-based marker of skeletal muscle mass in various thoracoabdominal surgeries. In Japan, sex-specific PMI cutoff values derived from healthy young Asian adults have been proposed and are commonly used in clinical research on sarcopenia and as indicators of whole-body skeletal muscle mass [15]. However, few and limited previous studies have investigated PPCs and prognosis post-esophagectomy using PMI as a preoperative muscle mass index in patients with esophageal cancer.

The iliopsoas muscles, including the psoas muscle, have distinct anatomical boundaries that support the high reproducibility of cross-sectional area and CT measurements [16]. In contrast, SMI measurements often include the multifidus and erector spinae muscles, which undergo significant fat infiltration with age [17]. Fat infiltration causes muscle heterogeneity and unclear boundaries. Accordingly, we aimed to compare the clinical utility of preoperative PMI- and SMI-based cutoff stratification in identifying patients at an increased risk of PPCs after esophagectomy for esophageal cancer. The primary endpoint was the occurrence of PPCs, and the secondary endpoints were 5-year OS and 5-year recurrence-free survival (RFS).

## Methods

### Patients and study design

The study was approved by the Committee for Ethics of the Kindai University School of Medicine (no. R06–015) and conducted in accordance with the ethical standards of the Declaration of Helsinki of 1964 and its amendments. Informed consent was obtained using an opt-out procedure, with study information disclosed on the hospital website; no patient declined to participate.

This study was conducted as a secondary analysis of a single-center retrospective cohort of patients with thoracic esophageal cancer who underwent esophagectomy at Kindai University Hospital between January 2016 and March 2024 [18]. The exclusion criteria were as follows: (a) two-stage surgery and (b) absence of preoperative CT examination.

## Data collection

The following data were retrospectively collected from electronic medical records: patient age, sex, comorbidities (chronic obstructive pulmonary disease [COPD], diabetes, heart failure), body mass index, pulmonary function (% predicted forced vital capacity [% pred. FVC] and % predicted forced expiratory volume [% pred. FEV_1_]), percentage of low-attenuation area (LAA%), prognostic nutrition index, smoking status, Brinkman index, preoperative treatment details (neoadjuvant therapy regimen), clinical stage, tumor localization, surgical information (thoracic and abdominal surgical modalities, lymphadenectomy, operation time, blood loss), and postoperative information (grade of PPCs, length of stay, postoperative prognosis). Variables were categorized using clinically meaningful cutoffs.

## Evaluation of PPCs

PPCs were defined as atelectasis or difficulty excreting sputum (need for bronchoscopy), pneumonia, initial ventilatory support for > 48 h, reintubation due to respiratory failure, or pneumothorax occurring within 2 weeks after esophagectomy [19]. Furthermore, all PPCs were graded using the Clavien‒Dindo classification, and events were considered present if the grade was ≥ II [20]. Medical record information from specific physicians in the surgery department was used to obtain details about PPCs diagnoses and procedures.

## SMI and PMI measurements

**Fig. 1:**
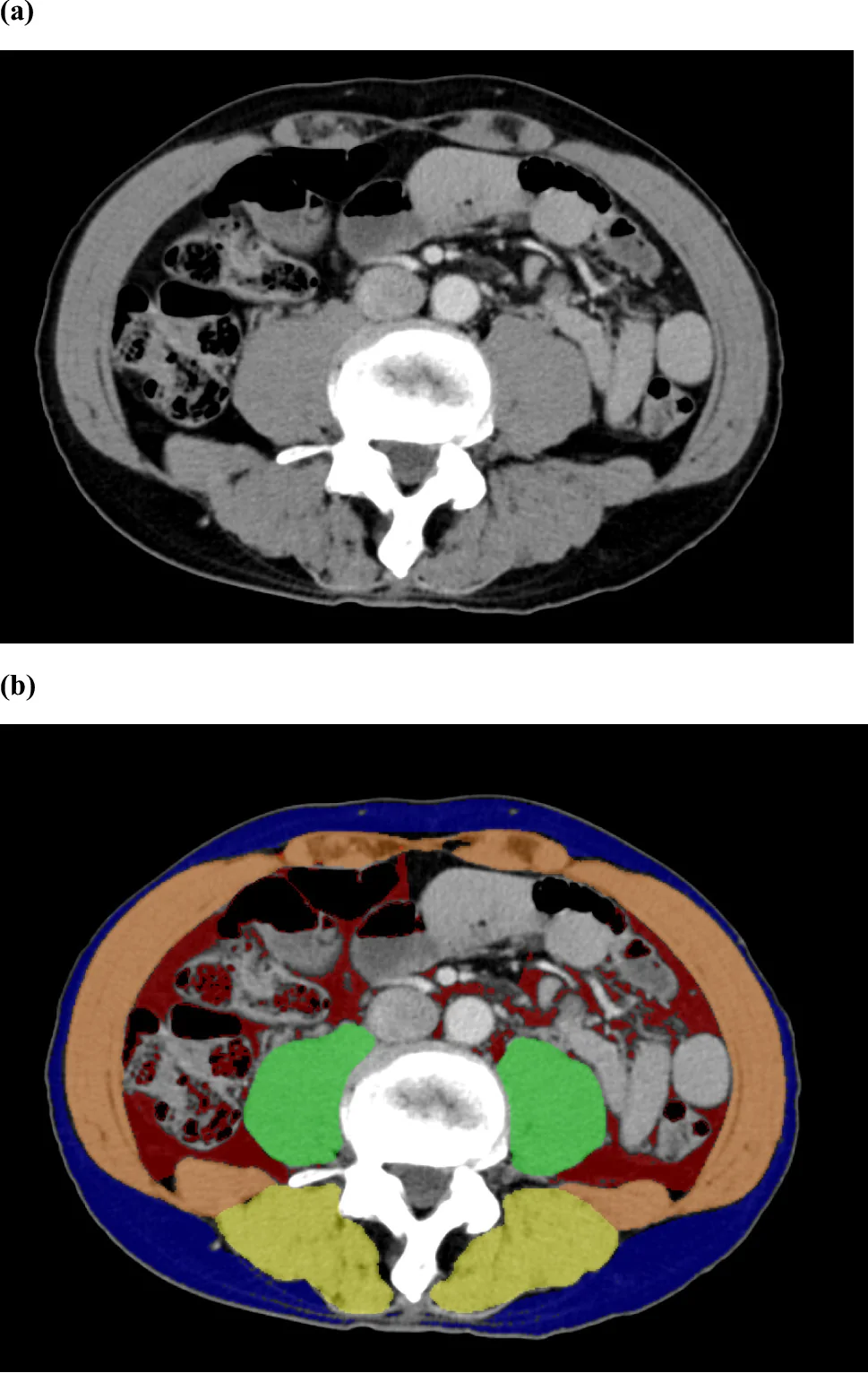
Representative axial CT images at the L3 level (**a**) Non-contrast CT image, (**b**) image at the time of analysi The sum of the orange, yellow, and green shaded areas indicates the cross-sectional skeletal muscle mass used to calculate the SMI, whereas the green shaded area alone corresponds to the psoas muscle mass used to calculate the PMI

The SYNAPSE VINCENT system (FUJIFILM Co., Ltd., Tokyo, Japan) and 5-mm CT slice images of the whole body were used to measure the SMI and PMI. CT images acquired within 1 month before esophagectomy were used for the analysis. The SYNAPSE VINCENT system automatically quantifies the skeletal muscle cross-sectional area based on Hounsfield units (from − 29 to 150). Briefly, the total skeletal muscle cross-sectional area and psoas muscle areas (cm^2^) were automatically measured at the level of the third lumbar vertebra (L3) (Fig. 1). After adjusting for patient height, SMI and PMI were calculated as the muscle area divided by the height squared. The SMI cutoff values were based on the report by Zhuang et al. [14] (men, 40.8 cm²/m²; women, 34.9 cm²/m²), because their values were derived from an Asian population with cancer and were thus considered more appropriate for our Japanese cohort than the Western-derived cutoff values. The PMI cutoff values were based on the sex-specific cutoff values proposed by Hamaguchi et al. [15] (men, 6.36 cm²/m²; women, 3.92 cm²/m²), which were derived from healthy Asian adults and have been widely adopted in Japanese clinical studies as computed tomography (CT)-based criteria for low skeletal muscle mass [21, 22]. Patients with SMI or PMI below the respective cutoff values were classified into the low-SMI or low-PMI groups, respectively.

### Statistical analysis

Data are presented as the means (standard deviations), medians (interquartile ranges [IQRs]), or numbers (percentages), as appropriate. Postoperative outcomes were compared between the groups using t-tests for normally distributed continuous variables, the Mann–Whitney U test for skewed continuous variables, and Fisher’s exact or χ² test for categorical variables. Univariate and multivariable logistic regression analyses were performed with PPCs as the dependent variable. The two skeletal muscle indices (low-SMI and low-PMI) were covariate-confounded with each other. Because SMI and PMI are both CT-based skeletal muscle indices and may be correlated with each other, two separate multivariable logistic regression models were constructed to avoid potential collinearity: Model I included Low-SMI, whereas Model II included low-PMI. Covariates for the multivariable logistic regression analyses were selected a priori based on clinical relevance and previous literature regarding risk factors for PPCs after esophagectomy, including patient demographics, body composition, pulmonary status, tumor stage, surgical approach, and nutritional status. These included age (per 1-year increase), sex (male vs. female), BMI (per 1-unit decrease), LAA% (per 1% increase), and% pred. FVC (per 1% decrease), clinical stage (per 1 stage increase), thoracotomy (yes or no), and PNI (per 1-unit decrease). We analyzed the differences in 5-year OS and 5-year RFS between the two groups divided according to PMI cutoff values using the Kaplan–Meier method and log-rank test. Patients with residual tumors (R1 resection) were excluded from the 5-year RFS event analysis. Cox proportional hazards regression analyses were performed to evaluate the associations of low SMI and low PMI with 5-year OS and 5-year RFS. To maintain consistency with the multivariable logistic regression analyses for PPCs, the Cox models were adjusted for the same covariates: age, sex, BMI, LAA%, and % pred. FVC, clinical stage, thoracotomy, and PNI. Statistical significance was set at *p* < 0.05. Statistical analyses were performed using R v.4.5.1 (R Foundation for Statistical Computing, Vienna, Austria) and EZR v.1.41 (Saitama Medical Center, Jichi Medical University, Saitama, Japan) software.

## Results

### Patient characteristics

**Fig. 2:**
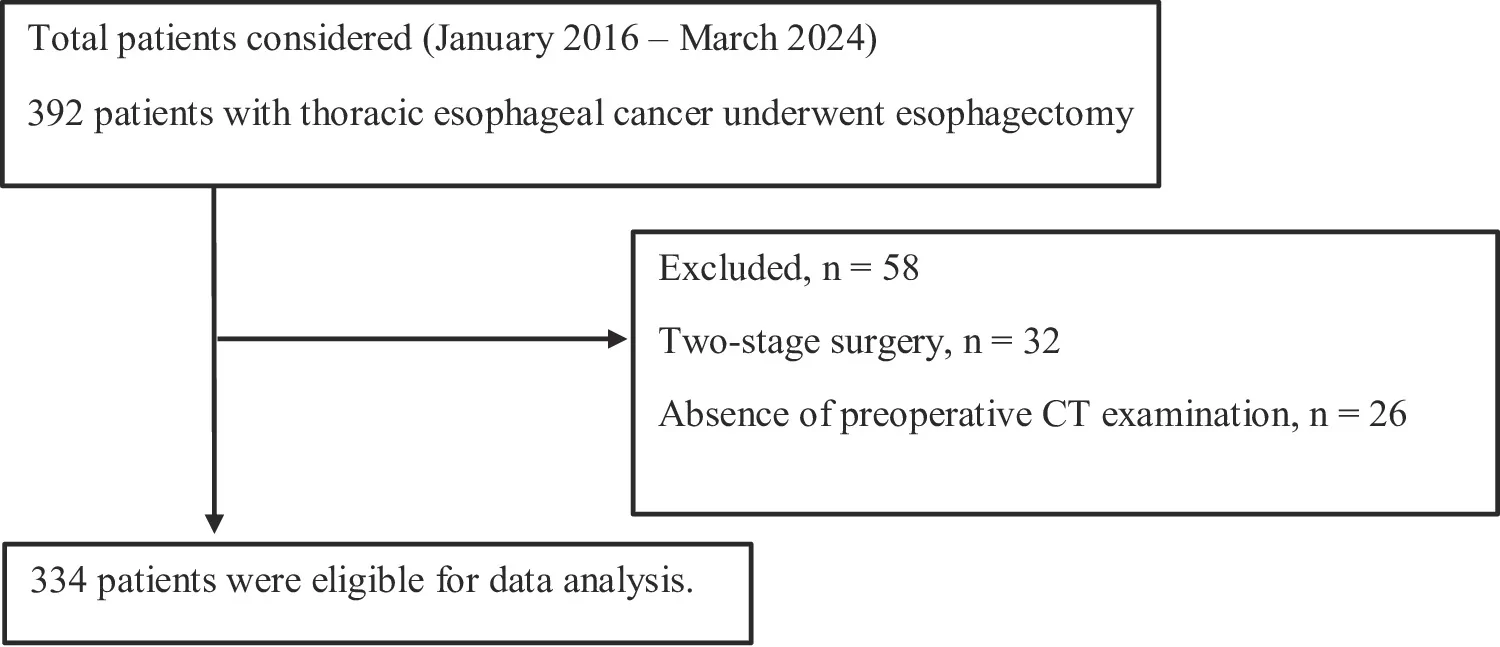
Flowchart of patient selection. In total, 392 patients with thoracic esophageal cancer who had undergone esophagectomy between January 2016 and March 2024 were considered for the analysis. We excluded 32 patients who underwent two-stage surgery and 26 patients who lacked a preoperative CT scan examination. Ultimately, 334 patients were included in the final analysis

Between January 2016 and March 2024, 392 patients with thoracic esophageal cancer underwent esophagectomy, of whom 32 underwent two-stage surgery. CT scans were not performed in 26 patients prior to esophagectomy; therefore, 58 patients with thoracic esophageal cancer were excluded, and 334 patients were included in the data analysis (Fig. 2). The patient characteristics are summarized in Table 1. The mean patient age was 69.2 ± 6.8 years, and 262 (78.4%) were male. Clinical stage III was the most common stage, and squamous cell carcinoma was the predominant histological type. The mean body mass index was 20.9 ± 3.2 kg/m², and the mean prognostic nutritional index was 45.4 ± 5.4. The mean SMI was 42.1 ± 7.6 cm²/m² for males and 34.9 ± 5.8 cm²/m² for females. The mean PMI was as follows: male, 5.7 ± 1.5 cm²/m² and female, 3.7 ± 1.1 cm²/m². Chronic obstructive pulmonary disease was present in 70 patients (21.0%). Neoadjuvant chemotherapy was administered to 248 patients (74.3%), and thoracotomy was performed in 222 patients (66.5%). Overall, PPCs occurred in 110 patients (32.9%), with pneumonia being the most frequent complication (21.6% of patients). The mean length of hospital stay was 44.7 ± 59.9 days.

**Table 1:** Baseline characteristics of patients with and without postoperative pulmonary complications

	All patients *n*=334
Age (years)	69.2±6.8
Male, n (%)	262 (78.4)
Clinical Stage I/II/III/IV	71/70/151/42
Cancer location: Upper/Middle/Low	50/140/144
SCC/AC/Other	298/21/15
Body Mass Index	20.9±3.2
Prognostic nutrition index	45.4±5.4
History of smoking, n (%)	264 (79.0)
Current Smoker, n (%)	136 (40.7)
Brinkman index	686.6±542.3
% pred. FVC (%)	97.2±14.7
% pred. FEV_1_ (%)	75.5±12.5
FEV_1_/FVC (%)	62.4±16.9
Skeletal muscle mass index (cm^2^/m^2^), male/female	42.1±7.6/34.9±5.8
Psoas muscle mass index (cm^2^/m^2^), male/female	5.7±1.5/3.7±1.1
Bilateral LAA%	0.8±2.4
Comorbidity	
Chronic obstructive pulmonary disease, n (%)	70 (21.0)
Diabetes, n (%)	42 (12.6)
Heart failure, n (%)	24 (7.2)
Neoadjuvant chemotherapy	
Chemotherapy, n (%)	248 (74.3)
Chemo-Radiation, n (%)	4 (1.2)
Surgical data	
Thoracotomy, n (%)	222 (66.5)
Thoracoscopy, n (%)	112 (33.5)
Hand-assisted laparoscopic surgery, n (%)	267 (79.9)
Reconstruction route	
Retrosternal/Subcutaneous/Posterior mediastinal	262/30/42
Reconstruction organ	
Stomach/Jejunum/others	303/27/4
Lymph nodes dissected	
2 fields/3 fields, n (%)	206 (61.7)/ 128 (38.3)
Duration of surgery (min)	687.7±110.1
Blood loss (mL)	540.3±421.9
Overall PPCs, n (%)	110 (32.9)
Pneumonia, n (%)	72 (21.6)
Atelectasis, n (%)	13 (3.9)
Difficulty excreting sputum, n (%)	6 (1.8)
Ventilatory support > 48 h or Reintubation, n (%)	7 (2.1)
Pneumothorax, n (%)	12 (3.6)
Length of Stay, (days)	44.7±59.9

All data are presented as mean ± standard deviation

AC: Adenocarcinoma, FEV1: Forced Expiratory Volume in 1 s, FVC: Forced Vital Capacity, LAA%: Low Attenuation Area Percentage, PPCs: Postoperative Pulmonary Complications, SCC: Squamous cell carcinoma

### Comparison of postoperative outcomes according to the SMI and PMI cutoffs

Table 2 shows that no significant differences were observed between the low-SMI and no low-SMI groups in the incidence of all-cause PPCs (34.7% vs. 31.5%, *p* = 0.56), pneumonia (21.2% vs. 22.0%, *p* = 0.89), atelectasis (4.3% vs. 3.3%, *p* = 0.78), difficulty excreting sputum (1.6% vs. 2.0%, *p* = 0.99), ventilatory support for > 48 h or reintubation (1.1% vs. 3.3%, *p* = 0.25), or pneumothorax (3.3% vs. 4.0%, *p* = 0.77). Length of hospital stay also did not differ significantly between the two groups (median, 32.0 days [IQR, 24.0–50.5] vs. 31.0 days [IQR, 23.8–45.3 days]; *p* = 0.34). In contrast, the low-PMI group had significantly higher incidences of all-cause PPCs (40.4% vs. 20.6%, *p* < 0.01) and pneumonia (27.4% vs. 11.9%, *p* < 0.01) than the no low-PMI group. However, no significant differences were observed between the two groups in the incidence of atelectasis (3.4% vs. 4.8%, *p* = 0.57), difficulty excreting sputum (2.4% vs. 0.8%, *p* = 0.42), ventilatory support for > 48 h or reintubation (2.4% vs. 1.6%, *p* = 0.71), or pneumothorax (4.8% vs. 1.6%, *p* = 0.22). Length of hospital stay was significantly longer in the low-PMI group than in the no low-PMI group (median, 33.0 days [IQR, 25.0–53.0] vs. 28.5 days [IQR, 22.0–43.8]; *p* = 0.02).

**Table 2:** Comparison of postoperative outcomes between groups stratified by the SMI and PMI cutoffs

	Low-SMI group *n* = 150	No low-SMI group *n* = 184	*p*-value	Low-PMI group *n* = 208	No low-PMI group *n* = 126	*p*-value
Overall PPCs, n (%)	52 (34.7)	58 (31.5)	0.56	84 (40.4%)	26 (20.6%)	<0.01
Pneumonia, n (%)	33 (21.2)	39 (22.0)	0.89	57 (27.4%)	15 (11.9%)	<0.01
Atelectasis, n (%)	5 (4.3)	8 (3.3)	0.78	7 (3.4%)	6 (4.8%)	0.57
Difficulty excreting sputum, n (%)	3 (1.6)	3(2.0)	0.99	5 (2.4%)	1 (0.8%)	0.42
Ventilatory support>48 h or Reintubation, n (%)	2 (1.1)	5 (3.3)	0.25	5 (2.4%)	2 (1.6%)	0.71
Pneumothorax, n (%)	6 (3.3)	6 (4.0)	0.77	10 (4.8%)	2 (1.6%)	0.22
Length of hospital stay, days, median (IQR)	32.0 (24.0–50.5)	31.0 (23.8–45.3)	0.34	33.0 (25.0–53.0)	28.5 (22.0–43.8)	0.02

Length of hospital stay was compared using the Mann–Whitney U test

Abbreviations: IQR, interquartile range; SMI, skeletal muscle index; PMI, psoas muscle index; PPCs, postoperative pulmonary complications

### Logistic regression analysis for PPCs after esophagectomy

Table 3 presents the results of the univariable and multivariable regression analyses of the risk factors for PPCs. In the univariable logistic regression analysis, a low SMI was not significantly associated with PPCs (odds ratio [OR] 1.15, 95% confidence interval [CI] 0.73–1.82; *p* = 0.54), whereas a low-PMI was significantly associated with PPCs (OR 2.61, 95% CI 1.56–4.35, *p* < 0.01). In the multivariable logistic regression analysis, a low SMI was not significantly associated with PPCs (OR 0.90, 95% CI 0.46–1.77; *p* = 0.76), whereas a low-PMI was significantly associated with PPCs (OR 2.17, 95% CI 1.16–4.07; *p* = 0.02). Because the present cohort was predominantly male, we performed an additional multivariable logistic regression analysis restricted to male participants only. In this analysis, a low PMI remained independently associated with PPCs (Model II: OR 2.07; 95% CI 1.10–3.89; *p* = 0.03), whereas a low SMI was not significantly associated with PPCs (Model I: OR 1.01; 95% CI 0.53–1.95; *p* = 0.97) (Supplementary Table 1).

**Table 3:** Univariable and multivariable analysis of risk factors for PPCs

	Univariable			Multivariable Model I: Low-SMI			Multivariable Model II: Low-PMI		
	OR	95% CI	*p*-value	OR	95% CI	*p*-value	OR	95% CI	*p*-value
Age (per 1 year increase)	1.07	1.04–1.11	<0.01	1.10	1.05–1.14	<0.01	1.09	1.05–1.14	<0.01
Sex (male vs. female)	3.37	1.69–6.71	<0.01	3.69	1.69–8.07	<0.01	2.97	1.35–6.53	<0.01
BMI (per 1 unit decrease)	1.03	0.96–1.11	0.44	0.96	0.86–1.08	0.48	1.02	0.93–1.13	0.66
LAA% (per 1% increase)	1.12	1.01–1.24	0.03	1.12	1.00–1.26	0.04	1.13	1.00–1.26	0.04
% pred. FVC (per 1% decrease)	1.01	0.99–1.02	0.30	1.01	0.99–1.03	0.31	1.01	0.99–1.03	0.36
Clinical stage (per 1 stage increase)	1.06	0.95–1.17	0.30	1.03	0.91–1.16	0.63	1.03	0.91–1.16	0.63
Thoracotomy (Yes vs. No)	2.16	1.28–3.63	<0.01	3.54	1.93–6.47	<0.01	3.66	1.99–6.74	<0.01
PNI (per 1 unit decrease)	1.03	0.99–1.07	0.21	1.01	0.96–1.06	0.75	1.00	0.95–1.05	0.93
Low-SMI (below cutoff value)	1.15	0.73–1.82	0.54	0.90	0.46–1.77	0.76	–	–	–
Low-PMI (below cutoff value)	2.61	1.56–4.35	<0.01	–	–	–	2.17	1.16–4.07	0.02

Abbreviations: BMI, body mass index; CI, confidence interval; LAA%, low attenuation area percentage; % pred. FVC, percent predicted forced vital capacity; PNI, prognostic nutritional index; SMI, skeletal muscle index; OR, odds ratio; PMI, psoas muscle index

### Postoperative prognosis after esophagectomy

The median follow-up duration was 36.9 months (interquartile range, 19.2–60.7). Regarding the 5-year OS, 98 patients died (5-year OS, 70.7%). In total, five patients had residual tumors (R1 resection); of these, four had values below the cutoff for both SMI and PMI, whereas the remaining one had values below the SMI cutoff but not the PMI cutoff.

**Fig. 3:**
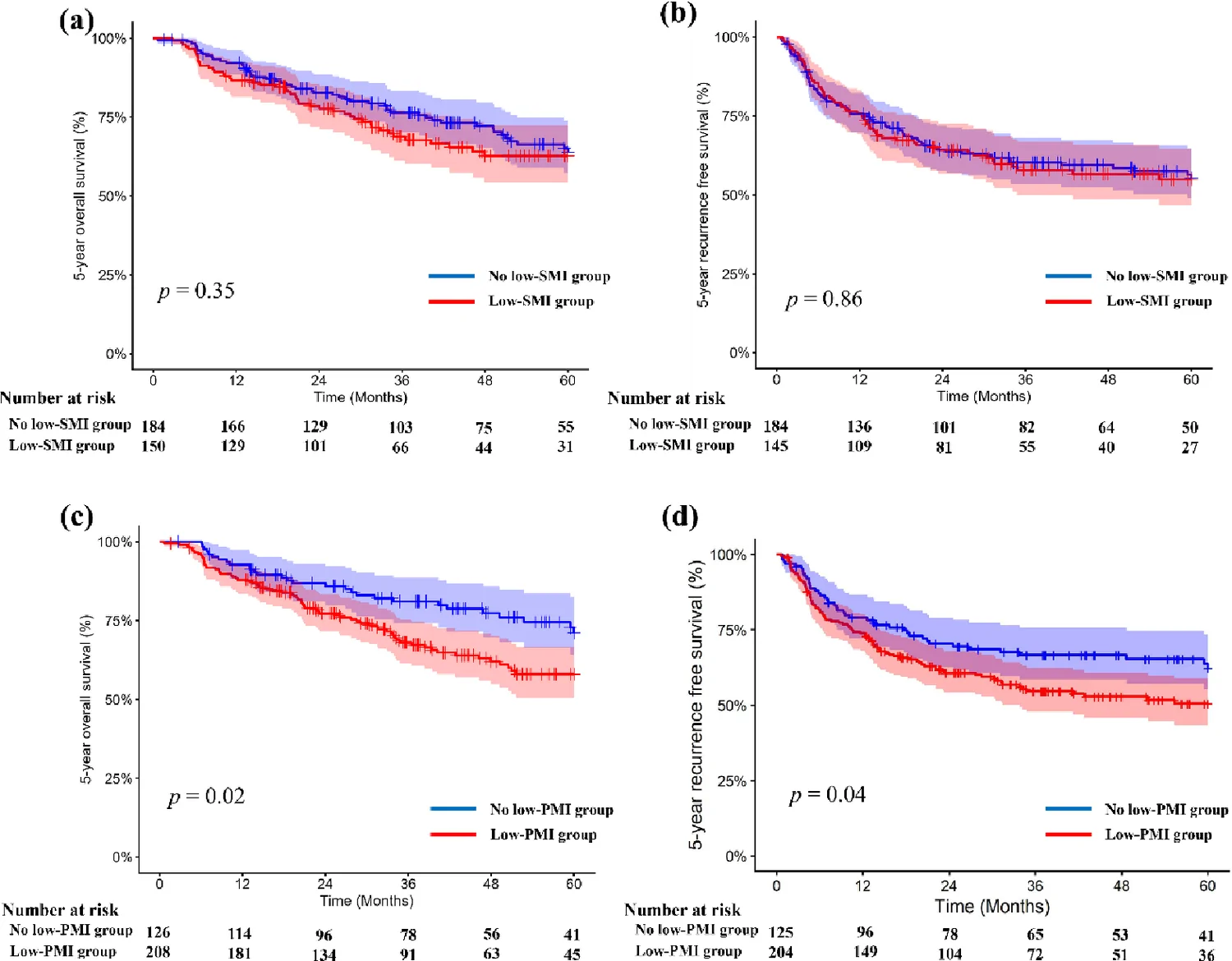
Kaplan–Meier curves for 5-year OS and RFS according to a low-SMI and a low-PMI status. (**a**) Comparison of 5-year OS between the no low-SMI group and the low-SMI group. (**b**) Comparison of 5-year RFS between the no low-SMI group and the low-SMI group. (**c**) Comparison of 5-year OS between the no low-PMI group and the low-PMI group. (**d**) Comparison of 5-year RFS between the no low-PMI group and the low-PMI group. The shaded areas represent the 95% confidence intervals, and the p-values were calculated using the log-rank test

In the no low group, 28 patients died (5-year OS, 77.8%), including 22 disease-specific deaths, five respiratory-related deaths, and one death from other causes (*n* = 1). In the low-PMI group, 70 patients died (5-year OS, 66.3%), comprising disease-specific deaths (*n* = 50), respiratory-related deaths (*n* = 14), and deaths from other causes (*n* = 6). The mortality rate differed significantly between the groups (*p* = 0.03). Between the no low-SMI and low-SMI groups, 5-year OS curves were comparable (log-rank *p* = 0.35; Fig. 3a), and no significant difference in RFS was observed (log-rank *p* = 0.86; Fig. 3b). In contrast, patients in the low-PMI group had significantly worse survival outcomes than those in the no low-PMI group; the low-PMI group showed significantly lower 5-year OS (log-rank *p* = 0.02; Fig. 3c) and significantly lower RFS (log-rank *p* = 0.04; Fig. 3d).

In the additional Cox proportional hazards regression analyses, the low-PMI group was significantly associated with worse 5-year OS in the univariable model (HR 1.67, 95% CI 1.07–2.62; *p* = 0.03). However, this association was not significant after adjusting for age, sex, BMI, LAA%, and% pred. FVC, clinical stage, thoracotomy, and PNI (HR 1.16, 95% CI 0.69–1.94; *p* = 0.58). Similarly, the low-PMI group was significantly associated with worse 5-year RFS in the univariable model (HR 1.59, 95% CI 1.08–2.33; *p* = 0.02), but this association was also attenuated after multivariable adjustment (HR 1.42, 95% CI 0.90–2.23; *p* = 0.13). Low SMI was not significantly associated with 5-year OS or 5-year RFS in either the univariable or multivariable analyses (Table 4). The detailed results, including all covariates, are presented in Supplementary Tables 2 and 3.

**Table 4:** Cox proportional hazards regression analyses for 5-year OS and RFS

Outcome	Index	Univariable			Multivariable		
		HR	95% CI	*p*-value	HR	95% CI	*p*-value
5-year OS	Low-SMI	1.23	0.82–1.82	0.31	0.86	0.50–1.48	0.59
	Low-PMI	1.67	1.07–2.62	0.03	1.16	0.69–1.94	0.58
5-year RFS	Low-SMI	1.03	0.73–1.45	0.86	0.90	0.58–1.41	0.65
	Low-PMI	1.59	1.08–2.33	0.02	1.42	0.90–2.23	0.13

Model I included Low-SMI, and Model II included Low-PMI. Each model was adjusted for age, sex, BMI, LAA%, % pred. FVC, clinical stage, thoracotomy, and PNI

Abbreviations: BMI, body mass index; CI, confidence interval; HR, hazard ratio; LAA%, low attenuation area percentage; OS, overall survival; PMI, psoas muscle index; PNI, prognostic nutritional index; % pred. FVC, percent predicted forced vital capacity; RFS, recurrence-free survival; SMI, skeletal muscle index

### Additional cutoff analyses

To examine the validity of the cutoff-based classification, additional sex-specific ROC analyses were performed using an institutional cohort. For PPCs, the AUCs for PMI were 0.584 in men and 0.557 in women, whereas those for SMI were 0.567 in men and 0.521 in women. The cohort-derived PMI cutoff for males was 5.96, which was relatively close to the prespecified cutoff used in the primary analysis. Time-dependent ROC analyses were also performed for the 5-year OS and RFS. The AUCs for 5-year OS ranged from 0.415 to 0.523, and those for 5-year RFS ranged from 0.401 to 0.566 (Supplementary Table 4).

## Discussion

In this single-center cohort of 334 patients who underwent esophagectomy, dichotomization using a prespecified PMI cutoff showed clear differences in pulmonary morbidity and survival between the groups. Patients in the low-PMI group had higher rates of overall PPCs and pneumonia than those in the no low-PMI groups. In the multivariable logistic regression analysis, a low PMI remained independently associated with PPCs, along with age ≥ 70 years, male sex, and thoracotomy. Complementary effect-scale analyses showed that a low PMI conferred a clinically meaningful increase in the absolute risk. Prognostically, 5-year OS and RFS were significantly lower in the low-PMI group (66.3%) than in the no low-PMI groups (77.8%), with a higher all-cause mortality rate. In unadjusted Kaplan–Meier and Cox analyses, the low-PMI group showed worse 5-year OS and RFS than the no low-PMI group. However, after adjusting for other prognostic factors in the Cox proportional hazards regression analyses, the association between a low PMI and long-term outcomes did not reach statistical significance. Therefore, the relationship between low PMI and long-term outcomes should be interpreted with caution. In contrast, a low PMI remained independently associated with PPCs in the multivariable logistic regression analysis, suggesting that the primary clinical relevance of PMI in this cohort may lie in its association with postoperative pulmonary morbidity. Collectively, these findings support the clinical utility of PMI for stratifying the risk of PPCs after esophagectomy, whereas its association with long-term outcomes requires further investigation.

Our results are consistent with those of prior studies showing that preoperative lumbar muscle-based indicators predict clinically significant risks in esophagectomy. Uemura et al. identified a low PMI as an independent predictor of major complications, with pneumonia being the most frequent event [23]. Mann et al. reported increased cardiopulmonary complications and worse OS in patients with low PMI [24]. Furthermore, Yunrong et al. found that a low PMI was associated with increased short-term mortality [25]. These data collectively support the potential of PMI to capture the vulnerability to PPCs and poor prognosis. The primary contribution of this study is the use of PMI and SMI metrics in pre-specified models and effect-size analyses. The PMI consistently stratified PPC risk and 5-year survival rates, whereas the SMI failed to show comparable independence or discriminatory ability, establishing the PMI’s comparative advantage. The PMI is defined as the cross-sectional area of the psoas muscles at a standardized vertebral level (usually L3) normalized by height squared [25, 26]. The PMI has been proposed as an inexpensive, simple, and validated surrogate for whole-body muscle mass [27]. We selected PMI as the muscle metric for several reasons. The iliopsoas muscles have distinct anatomical boundaries, whereas SMI measurements often include the multifidus and erector spinae muscles, which undergo significant fat infiltration with age [16, 17]. Shichinohe et al. reported that in patients with esophageal cancer, total muscle mass can be inferred through PMI measurement alone, given the high correlation between the psoas and aggregate muscle areas [22]. Therefore, PMI is an attractive and efficient marker for routine risk assessment, especially in busy clinical settings, where full-body composition analysis may be impractical. However, these findings should not be interpreted as demonstrating the true biological superiority of PMI over SMI. Both PMI and SMI are CT-based muscle area indices measured at the L3 level, with SMI reflecting the total skeletal muscle cross-sectional area and PMI reflecting the psoas muscle area. Neither index accounted for muscle quality, myosteatosis, functional status, or whole-body skeletal muscle mass assessed using bioelectrical impedance analysis or dual-energy X-ray absorptiometry. Therefore, the stronger association observed for PMI may partly reflect differences in the selected L3-level area-based definitions and their methodological characteristics rather than an inherent PMI superiority.

Several issues regarding cutoff-based classification should be considered when interpreting these findings. The SMI cutoff used in this study was originally derived from a gastric cancer cohort, whereas the PMI cutoff was derived from adult donors undergoing living-donor liver transplantation. Therefore, these cutoff values may not be fully optimized for patients undergoing esophagectomy due to esophageal cancer. In particular, the negative findings for SMI may partly reflect the limitations of the selected cutoff values rather than a lack of clinical relevance of SMI itself. Our cohort-derived cutoffs showed only modest discriminative ability for PPCs, and time-dependent ROC analyses showed limited predictive ability for 5-year OS and RFS. These results suggest that deriving new cutoffs from the present cohort is unlikely to substantially improve risk discrimination and could increase the risk of overfitting, particularly in sex-specific analyses. Therefore, we retained the prespecified cutoffs for the main analysis. Nevertheless, these findings highlight the need for multicenter studies to establish esophagectomy-specific cutoff values for SMI and PMI.

Multiple mechanisms likely underlie the association between low PMI and postoperative outcomes. We assumed that sarcopenia, frailty, and respiratory muscle weakness were the central considerations. Sarcopenia, a core component of frailty, encompasses the loss of skeletal muscle mass and strength [28–30]. Diminished muscle mass directly translates to weaker respiratory muscles, including the diaphragm, intercostals, and accessory respiratory muscles [31]. Previous reports have shown that peak cough flow is significantly associated only with respiratory muscle strength; furthermore, peripheral muscle strength is associated with respiratory muscle strength and peak cough flow [32]. These findings support the hypothesis that sarcopenia or respiratory muscle weakness leads to atelectasis, secretion retention, and ultimately pneumonia [33, 34]. In our cohort, we suspected that patients with a low PMI had subclinical respiratory impairment (lower % pred). FVC, as evidenced by their significantly higher rate of pneumonia and need for ventilatory support. Furthermore, PPC development in patients post-esophagectomy is associated with poor postoperative prognosis [35]. Our findings are consistent with those of previous studies linking PPCs to reduced survival after esophagectomy. These findings support a perioperative strategy that couples the preoperative identification of patients with a low PMI with targeted prehabilitation. A recent study on esophageal cancer suggested that structured prehabilitation during neoadjuvant therapy can preserve or even improve body composition relative to usual care and may mitigate typical treatment-related muscle loss, potentially lowering PPC risk and supporting recovery trajectories [36]. Incorporating PMI-guided referral thresholds into perioperative pathways, combined with respiratory-focused training and nutritional optimization, represents a testable next step for improving pulmonary outcomes and survival in this high-risk population.

This study has several limitations. First, it was a single-center retrospective analysis, and surgical techniques and perioperative management protocols vary among institutions; therefore, our findings reflect the practices of only one institution. The present cohort was predominantly male, and male sex was independently associated with PPCs. Although a low PMI remained independently associated with PPCs in an additional multivariable analysis restricted to male patients, the relatively small number of female patients limited the evaluation of sex-specific associations. Second, the enrollment period was relatively long, extending from January 2016 to March 2024. Surgical techniques, neoadjuvant treatment strategies, and perioperative management have evolved over this period, potentially influencing the incidence of PPCs and long-term survival outcomes. Although thoracotomy and clinical stage were included as covariates in the multivariable models, residual confounding related to temporal changes could not be excluded. Third, muscle quality and functional status were not assessed; therefore, some patients with frailty may have been missed. External validation in diverse cohorts is required to confirm the generalizability of these findings.

## Conclusions

In conclusion, a preoperative low PMI was independently associated with higher PPC rates, particularly pneumonia, whereas preoperative SMI-based stratification was not significantly associated with PPCs. Although a low PMI was associated with worse 5-year OS and RFS, these associations were not independent of other prognostic factors. These findings suggest that the preoperative PMI may be a simple CT-based marker of postoperative pulmonary morbidity after esophagectomy, although its relationship with long-term outcomes requires cautious interpretation and further validation in future studies.

## Supplementary Information

Below is the link to the electronic supplementary material.

Supplementary Material 1

## Data Availability

All data generated or analyzed in this study are included in this article. Further inquiries should be directed to the corresponding author.
