# Supplementary material for: Comparative clinical utility of preoperative psoas muscle and skeletal muscle index cutoffs for risk stratification after esophagectomy
Source: Surg Today. 2026 Jul 23;56(11):2836–45. doi: 10.1007/s00595-026-03433-7 (PMC13722738; doi:10.1007/s00595-026-03433-7)
Supplement: Supplementary file 1 [file 595_2026_3433_MOESM1_ESM.docx]

**R1_SUPPLEMENTARY TABLES**

**Table S1.** Multivariable logistic regression analyses for PPCs, restricted to male patients.

|  |  | Multivariable  Model I: Low-SMI | | |  | Multivariable  Model II: Low-PMI | | |
| --- | --- | --- | --- | --- | --- | --- | --- | --- |
|  |  | OR | 95% CI | *p*-value |  | OR | 95% CI | *p*-value |
| Age (per 1 year increase) | | 1.09 | 1.05–1.14 | <0.01 | | 1.09 | 1.05–1.13 | <0.01 |
| BMI (per 1 unit decrease) | | 0.99 | 0.89–1.10 | 0.90 | | 0.95 | 0.87–1.04 | 0.29 |
| LAA% (per 1% increase) | | 1.13 | 1.02–1.26 | 0.03 | | 1.13 | 1.01–1.26 | 0.03 |
| % pred. FVC  (per 1% decrease) | | 1.01 | 0.99–1.03 | 0.30 | | 1.01 | 0.99–1.03 | 0.37 |
| Clinical stage  (per 1 stage increase) | | 1.05 | 0.93–1.18 | 0.43 | | 1.04 | 0.92–1.17 | 0.53 |
| Thoracotomy (Yes vs No) | | 3.58 | 1.99–6.47 | <0.01 | | 3.81 | 2.10–6.93 | <0.01 |
| PNI (per 1 unit decrease) | | 1.02 | 0.97–1.07 | 0.52 | | 1.01 | 0.96–1.06 | 0.65 |
|  | |  |  |  | |  |  |  |
| Low-SMI (below cutoff value) | | 1.01 | 0.53–1.95 | 0.97 | | – | – | – |
| Low-PMI (below cutoff value) | | – | – | – | | 2.07 | 1.10–3.89 | 0.03 |

Model I included low SMI, and Model II included low PMI. Each multivariable logistic regression model was adjusted for age, BMI, LAA%, % pred. FVC, clinical stage, thoracotomy, and PNI. Sex was not included in the male-only analysis.

Abbreviations:

BMI, body mass index; CI, confidence interval; LAA%, low attenuation area percentage; OR, odds ratio; PMI, psoas muscle index; PNI, prognostic nutritional index; PPCs, postoperative pulmonary complications; % pred. FVC, percent predicted forced vital capacity; SMI, skeletal muscle index.

**Table S2.** Univariable and multivariable Cox proportional hazards regression analyses for 5-year OS

|  |  | Univariable | | |  | Multivariable  Model I: Low-SMI | | |  | Multivariable  Model II: Low-PMI | | |
| --- | --- | --- | --- | --- | --- | --- | --- | --- | --- | --- | --- | --- |
|  |  | HR | 95% CI | *p*-value |  | HR | 95% CI | *p*-value |  | HR | 95% CI | *p*-value |
| Age (per 1 year increase) | | 1.02 | 0.99–1.04 | 0.25 | | 1.02 | 0.99–1.05 | 0.16 | | 1.02 | 0.99–1.05 | 0.18 |
| Sex (male vs female) | | 1.72 | 0.98–3.03 | 0.06 | | 1.67 | 0.91–3.09 | 0.10 | | 1.61 | 0.87–2.98 | 0.13 |
| BMI (per 1 unit decrease) | | 1.05 | 0.98–1.12 | 0.14 | | 1.06 | 0.96–1.17 | 0.24 | | 1.03 | 0.95–1.12 | 0.46 |
| LAA% (per 1% increase) | | 1.06 | 0.99–1.13 | 0.11 | | 1.03 | 0.94–1.12 | 0.60 | | 1.02 | 0.94–1.12 | 0.60 |
| % pred. FVC  (per 1% decrease) | | 1.01 | 0.99–1.03 | 0.06 | | 1.01 | 0.99–1.03 | 0.14 | | 1.01 | 1.00–1.03 | 0.16 |
| Clinical stage  (per 1 stage increase) | | 1.25 | 1.14–1.38 | <0.01 | | 1.24 | 1.12–1.37 | <0.01 | | 1.24 | 1.12–1.37 | <0.01 |
| Thoracotomy (Yes vs No) | | 1.24 | 1.76–2.02 | 0.39 | | 1.34 | 0.80–2.23 | 0.27 | | 1.35 | 0.81–2.25 | 0.25 |
| PNI (per 1 unit decrease) | | 1.05 | 1.01–1.09 | <0.01 | | 1.03 | 0.99–1.07 | 0.12 | | 1.03 | 0.99–1.07 | 0.14 |
|  | |  |  |  | |  |  |  | |  |  |  |
| Low-SMI (below cutoff value) | | 1.23 | 0.82–1.82 | 0.32 | | 0.86 | 0.50–1.48 | 0.59 | | – | – | – |
| Low-PMI (below cutoff value) | | 1.67 | 1.07–2.62 | 0.03 | | – | – | – | | 1.16 | 0.69–1.94 | 0.58 |

Model I included low SMI, and Model II included low PMI. Each multivariable Cox proportional hazards model was adjusted for age, sex, BMI, LAA%, % pred. FVC, clinical stage, thoracotomy, and PNI.

Abbreviations: BMI, body mass index; CI, confidence interval; HR, hazard ratio; LAA%, low attenuation area percentage; OS, overall survival; PMI, psoas muscle index; PNI, prognostic nutritional index; % pred. FVC, percent predicted forced vital capacity; SMI, skeletal muscle index.

**Table S3.** Univariable and multivariable Cox proportional hazards regression analyses for 5-year RFS

|  |  | Univariable | | |  | Multivariable  Model I: Low-SMI | | |  | Multivariable  Model II: Low-PMI | | |
| --- | --- | --- | --- | --- | --- | --- | --- | --- | --- | --- | --- | --- |
|  |  | OR | 95% CI | *p*-value |  | OR | 95% CI | *p*-value |  | OR | 95% CI | *p*-value |
| Age (per 1 year increase) | | 1.01 | 0.99–1.04 | 0.24 | | 1.02 | 0.99–1.04 | 0.18 | | 1.01 | 0.99–1.04 | 0.27 |
| Sex (male vs female) | | 1.56 | 0.98–2.49 | 0.06 | | 1.42 | 0.86–2.34 | 0.17 | | 1.33 | 0.80–2.20 | 0.27 |
| BMI (per 1 unit decrease) | | 1.00 | 0.95–1.06 | 0.92 | | 1.00 | 0.93–1.08 | 0.99 | | 0.96 | 0.90–1.03 | 0.30 |
| LAA% (per 1% increase) | | 1.06 | 1.01–1.12 | 0.03 | | 1.05 | 0.99–1.12 | 0.13 | | 1.05 | 0.98–1.11 | 0.15 |
| % pred. FVC  (per 1% decrease) | | 1.05 | 0.99–1.02 | 0.39 | | 1.00 | 0.99–1.02 | 0.59 | | 1.00 | 0.99–1.02 | 0.65 |
| Clinical stage  (per 1 stage increase) | | 1.26 | 1.16–1.36 | <0.01 | | 1.25 | 1.15–1.36 | <0.01 | | 1.25 | 1.14–1.36 | <0.01 |
| Thoracotomy (Yes vs No) | | 0.98 | 0.67–1.24 | 0.89 | | 1.06 | 0.72–1.57 | 0.77 | | 1.08 | 0.73–2.56 | 0.71 |
| PNI (per 1 unit decrease) | | 1.03 | 1.00–1.06 | 0.04 | | 1.01 | 0.98–1.05 | 0.43 | | 1.01 | 0.98–1.05 | 0.46 |
|  | |  |  |  | |  |  |  | |  |  |  |
| Low-SMI (below cutoff value) | | 1.03 | 0.73–1.45 | 0.86 | | 0.90 | 0.58–1.41 | 0.65 | | – | – | – |
| Low-PMI (below cutoff value) | | 1.59 | 1.08–2.33 | 0.02 | | – | – | – | | 1.42 | 0.90–2.23 | 0.13 |

Model I included low SMI, and Model II included low PMI. Each multivariable Cox proportional hazards model was adjusted for age, sex, BMI, LAA%, % pred. FVC, clinical stage, thoracotomy, and PNI.

Abbreviations: BMI, body mass index; CI, confidence interval; HR, hazard ratio; LAA%, low attenuation area percentage; PMI, psoas muscle index; PNI, prognostic nutritional index; % pred. FVC, percent predicted forced vital capacity; RFS, recurrence-free survival; SMI, skeletal muscle index.

**Table S4.** Additional ROC and time-dependent ROC analyses for cohort-derived cutoff values of PMI and SMI

| **Outcome** | **Index** | **Sex** | **Cutoff value** | **AUC** | **Sensitivity** | **Specificity** |
| --- | --- | --- | --- | --- | --- | --- |
| PPCs | SMI | Male | 42.62 | 0.567 | 0.521 | 0.616 |
|  |  | Female | 32.55 | 0.521 | 0.623 | 0.545 |
|  | PMI | Male | 5.96 | 0.584 | 0.485 | 0.707 |
|  |  | Female | 1.78 | 0.557 | 1.000 | 0.182 |
|  |  |  |  |  |  |  |
| 5-year OS | SMI | Male | 43.54 | 0.451 | 0.427 | 0.598 |
|  |  | Female | 31.77 | 0.497 | 0.874 | 0.343 |
|  | PMI | Male | 2.71 | 0.415 | 0.997 | 0.029 |
|  |  | Female | 3.01 | 0.523 | 0.867 | 0.304 |
|  |  |  |  |  |  |  |
| 5-year RFS | SMI | Male | 46.72 | 0.467 | 0.274 | 0.783 |
|  |  | Female | 31.77 | 0.566 | 0.925 | 0.412 |
|  | PMI | Male | 2.71 | 0.401 | 0.991 | 0.029 |
|  |  | Female | 3.01 | 0.534 | 0.879 | 0.343 |

ROC analyses were performed for PPCs, and time-dependent ROC analyses were performed for 5-year OS and 5-year RFS. Cutoff values were derived from the present institutional cohort.

Abbreviations: AUC, area under the curve; OS, overall survival; PMI, psoas muscle index; PPCs, postoperative pulmonary complications; RFS, recurrence-free survival; ROC, receiver operating characteristic; SMI, skeletal muscle index.
